# Editorial

**Published:** 1876-09

**Authors:** 


### Editorial.

### QUACKERY.

Quack, "a boastful pretender to medical skill; one who boastfully pretends to knowledge not possessed; an ignorant and pretentious practitioner in any branch of knowledge; a charlatan; a mountebank,"

Where and how does quackery flourish, and by whom is it aided and fostered?

The almost universal reply to the first point of this question would be, *Everywhere.* There is scarcely an avocation of human life in which it is not found, to a greater or less extent; but more perhaps in the professional and scientific occupations than elsewhere; there seems to be in these more scope for pretentious, boastful and hypothetical assumption.

And as every one sees more distinctly in his own immediate territory, there seems to us, more of this obnoxious and hateful thing called quackery in the dental profession than any where else. We have, however, some times heard the practitioner of general medicine affirm, that no where is there more charlatanism to be found than in the medical profession. On this point we are not disposed to make controversy, but will rest content with what we have, and if any other profession is cursed with more, then we will try and exercise a larger measure of sympathy than heretofore.

Measuring the dental profession by the scale of popular respectability, quackery exists from the top to the bottom; it does not taper to a point at the top either, and it is immensely broad at the bottom.

Measuring by the scale of attainments, quackery extends very far towards the top, and is almost co-extensive with the profession itself at the base.

Quackery is a tree that bears fruit luxuriantly; late frosts never kill it; heat never blights it; mildew never strikes it; floods never drown it; insects never destroy it, It will flourish under almost any condition, except under the clear, shining rays of bright *light*; under this it withers and is destroyed.

Quackery flourishes in dark, damp soil. Ignorance is its fertilizer.

By whom is quackery encouraged and aided? A very important question truly. In searching for replies to this question, we will begin at home.

First, it is aided by all those members of the profession who encourage young men, who have no natural or acquired ability, to enter upon a course of dental study.

Second, by all those who take students with the promise to fit them for practice in a few weeks, months or a year at most.

Third, by all those who instill into the minds of students, meager or false conceptions and ideas in reference to the preparation requisite for dental practice, or imperfect and degraded views of the practice itself.

Fourth, by those who themselves go, in their practice, just as near the boundary of quack territory as possible; this is often done in modes of practice, and in professional business affairs—such as making unwarranted claims, and assuming superior ability, and advertising in a questionable way.

Fifth, quackery is aided by the people, when they, either from ignorance or avarice, refuse or fail to discriminate between the honorable, well qualified and efficient practitioner, and the incompetent charlatan; and in estimation place them upon a level, or, as is too often the case, give preference to the latter. This in, perhaps, a majority of instances, occurs from ignorance, inability to judge of human nature, or the professional qualifications of him whose service they seek; and oftentimes from an almost total want of appreciation of their teeth,

Sixth, quackery is encouraged by physicians when they assert that dentistry is nothing more than a mechanical art, and that any one who is skillful with instruments can be a good dentist, and that it is wholly embodied in manipulation. And again, when they assert that a knowledge of the fundamental principles of medical science is wholly superfluous. Upon this ground only can the fact be explained, that physicians are frequently as easily imposed upon by quack dentists as any other class of persons; and they will sometimes espouse the cause of the most consumate charlatan, and bear him upon their wings to at least a temporary success. Eminent physicians have done and are doing just such things.

Seventh, Ministers have far too often manifested "hot haste" to give their endorsement and recommendation to the arrant quack, Altogether too frequently have we seen the name of Rev. A. B., and C. D., and E. F. certifying to qualifications about which they know nothing, and to the success of a dental vandal. Usually the people place so much reliance in the opinions of the physician and the minister, that their recommendation of that about which they know nothing, inspires more confidence than the statement of most other persons based on absolute knowledge.

Now the existence of this confidence is not chargeable to the physician or the minister, but when they happen to be mistaken, or make wrong statements about that of which they are totally ignorant, it is hard for the victims.

Eighth. The lawyers! No; they are usualy good judges of human nature, and are not liable to be caught by quacks, at least to the extent of vouching for and endorsing them. We have in a few instances, however, seen the name of a judge, or govenor, or colonel appended to some charlatan advertisement; but they are now very rare.

Quacks in the legal profession are few compared with the number in the different departments of the medical profession. When the people wish care and protection for their money and property, they will not tolerate charlatanism and incompetency for one moment, but will scrutinize and investigate closely as to honesty and ability. But it is quite a different matter when restoration and care for the health of the body is in question. Any body who will make the largest promises, and the smallest fees, is too often the one to be secured.

Ninth. Very great aid, support and encouragement is given to quacks by the editorial fraternity. The press is most basely prostituted in this respect. By common consent the *secular* press, without "let or hindrance," advertises all kinds of quackery that will pay. It is generally regarded as *business*, one into which no moral or humanitarian element enters; it is too often simply to make money for the press and the quack, out of the victims of the latter, and too often in the transaction, is involved the loss of health, and frequently loss of life. The most consumate empiric may prepare matter in the form of an advertisement, consisting simply of bombastic egotism, assumption, falsehood and slander, and he will have no difficulty in finding those who will proclaim it to twenty or fifty thousand people every day, so long as it is paid for. The press does not publish *every thing* in the form of advertisements that is presented, but they all publish by far too much. How much curtailment would there be in the advertisements of quacks and nostrums, if the question was always asked, Is this true? If not it must be rejected. However the secular press may be regarded in this matter, it does seem but just and reasonable that the religious press which assumes, to some extent at least, to give direction in moral and religious affairs, and to seem in these particulars as educators, should discriminate against falsehood and the usually accompanying abominations, in their advertising departments.

A glance at some of the most popular religious newspapers in the country will satisfy any one that the most gigantic frauds, swindles and charlatanry are put forth with all the plausibility of the most sacred truth, In this matter we must mention one exception. The *New York Observer* has this announcement at the head of its advertising columes: "*The Observer refuses advertisements of medicines, of bad books, and of every thing that tends to the injury of the community.*" Now we dislike to be partial, but so far as we are aware, no other paper has given authority for the same kind of a statement. We want the *Observer*. Now we have directed attention to a few, and only a few, of the ways by which quackery in our profession is sustained, and we hope others will take up the subject and make thorough work, regardless as to where the blows fall, or how deep they bruise.

### EDUCATIONAL—STUDENTS.

The time for the assembling of the hosts to the various educational institutions throughout the land is near at hand. Anxiety and solicitude is, no doubt, entertained by most, if not all interested.

The student is solicitous in respect to his entrance, to his progress, and especially to his outcome.

It has been our lot to have cognizance of students seeking to enter the dental profession. They are, perhaps, in many respects, similar to students seeking to enter other professions. It is to be remembered, however, that law, general medicine and theology, as professions, have by age, attained a permanency of plans and operations in educational matters, that can not attach to any profession of so recent growth as dentistry, and as a rule students are but too ready to seek to take advantage of this fact, and in a variety of ways. And in the first place they generally imagine that a less amount of money is necessary for a course of instruction in dentistry than is required in any other profession, a very circumscribed and meager conception of what a perfectcourse of dental education involves. This is either from pureignorance or from false notionsimbibed from defective teaching.

Again, students desire to complete their course in the shortest time. The demand for graduation in the shortest possible time, is often pressed with great urgency. Too many imagine that if graduation is attained the goal is won—success is certain. We would suggest to all such, that a diploma obtained without the proper attainments as a basis, is got at a fearful cost.

Students will sometimes dicker between colleges for terms, early graduation, etc., etc. In a letter just at hand, one says: "I must graduate with one course of lectures. I have a proposition from another college to that effect, and if you can not promise that I shall so graduate, I will go there, but I had rather go to your college." Another says, "I want to go to your college, but I must have six months time on my tuition fee. Another college has agreed to accept my proposition, and if you do not I must go there." This gentleman is an entire stranger to us, and offers no security or evidence of responsibility. To all such we reply, go to the "other college."

Another says, "If you don't in my case remit some of the branches of study in your curriculum, I must go elsewhere. I must go through in one course, and I don't think I could do justice to all the branches in that time. I hope you will consent to omit in my case, two of the branches." He names them. This was very kind. Of course no such concession could be made. To these many more might be added, but they are sufficiently representative to indicate the chaffering that is often resorted to. The tendency of such things is demoralizing, unless promptly and sharply resented.

The presumption is, that every college devises such rules, regulations and course of instruction as is for the best interest of the student, the profession of which he is to become a member, and the people whom he is to serve; and these should be scrupulously observed, otherwise demoralization and inefficiency will necessarily creep in.

Now our readers will perceive that dental colleges are subjected to annoyances that ought not to exist. Students would not for a moment think of making such demands of law, medical or literary colleges.

Such a spirit as is indicated by the above extracts is evidence, to some extent at least, of unfitness even to enter college. Let such rules and regulations be instituted in all our colleges, as shall serve the highest interest of the profession and the public, and then let them be inflexibly observed.

### A LADY D. D. S.

It was recently our privilege to call upon and make the acquaintance of Miss A. Ramberger, D. D. S., of Philadelphia. She graduated one year ago in the Pennsylvania Dental College; occupying an enviable position in her class. We found her in her office, a very neat and convenient one, quite at home, without embarrassment, and appearing quite as natural in her novel position, as any one of the male persuasion. As to practice, she is making a splendid success, operating only for ladies and children—will not touch one of *us*; this seems pretty hard, but she knows best. As to her operations every one, having knowledge of the same reported them as excellent.

She of course had some prejudice to meet, and overcome, but she *can* and *is* doing it, and we do not hesitate to predict for her a noble and useful career; we only wish there were in our country, a thousand more like her; there is room, yes more, a great demand. Three fourths of all the operations upon the natural teeth of ladies and children, could be just as well done, and in a great many cases far better, by lady operators, than by men; let the latter haggle away upon one another, but women understand and appreciate the requirements, of their own delicate and sensitive organigations and that of children as well, better than men can do. All success to Miss R,

### OBITUARY.

Died suddenly, on July 25th, 1876, Dr. Elias Wildman, M. D., D. D. S., in the sixty-sixth year of his age.

His death will occasion surprise and sorrow throughout the dental profession.

We have known him for many years, but though not so intimately as some others, yet we knew him well enough, to regard and esteem him very highly indeed. He possessed some admirable qualities that are rarely found in these days. Dr. Wildman was best esteemed, appreciated and loved by those who knew him best.

The following we take from an advance sheet of the *Dental Cosmos* for Sept.

"Dr. Wildman was born near Morrisville, Bucks County Pa., June 8th, 1811; graduated at the University of Pennsylvania 1833; practiced medicine for a short time in Bucks County; served in hospital practice in New York City for some months; studied dentistry with the brothers Birkey, and commenced its practice in 1836. He was elected a professor in the Pennsylvania College of Dental Surgery in June, 1862, and was made dean in 1871. The latter position he resigned in the spring of the present year, but held the chair of Mechanical Dentistry and Metallurgy up to the time of his death.

In the space to which we are necessarily limited it is simply impossible to do justice to the character and attainments of Professor Wildman; but we should do violence alike to our own feelings and to those of our readers if we did not place on record in this journal, devoted to the interests of the profession with which he was so long identified, our estimate of his worth and our sense of its loss.

Dr. Wildman was of such an unaffectedly diffident disposition that his merits were never properly appreciated, except by those who had known him long and intimately. He was not calculated nor disposed to attract attention to himself, and never appeared to so much advantage as when engaged in his laboratory in the investigation of some difficult problem. Under such circumstances his industry was remarkable and his patience inexhaustible.

In 1836—the same year in which he commenced the practice of dentistry—he began experimenting in the manufacture of porcelain teeth. He entered upon the work with great determination, and continued it with unflagging zeal until his efforts were crowned with a success far beyond that of any previous experimenter. He achieved results in the translucency and life-like appearance of his products which had not previously been obtained and have not since been excelled. His most notable success was in the methods by which it became possible to secure uniformity of results in the gum-enamel. After an immense number of experiments he succeeded, in 1839, in obtaining a gum-enamel which has not been surpassed to the present day, as specimens of his work done in that year are still in existence to demonstrate. To Dr. Wildman must be accorded the honor of having been the first to reduce the manufacture of porcelain teeth to a scientific basis.

His experiments with rubber were equally thorough. The results of these were first published in 1865, under the title of "Instructions in the Manipulation of Hard Rubber or Vulcanite for Dental Purposes." This volume was so much sought for that six editions of it were disposed of.

Not less exhaustive was his experimentation with celluloid. Having visited the factory, and learned by conversation and observation all that could be gathered there relative to materials, processes, and results, he brought home with him specimens of the substance in all its stages. With these and with the finished product he made many and ingenious experiments to determine its properties. It is safe to say that Dr. Wildman probably knew as much of celluloid in its application to dental purposes as any other living man.

These are but illustrations of the manner in which he conducted his investigations in many other directions. A careful observer, a most methodical and exact experimenter, he never jumped at conclusions in the pursuit of information, but arrived at them as the result of plodding, painstaking, persistent investigation, of observation, comparison, reflection and the record of successive steps or stages in the work.

As a teacher, he was thorough, conscientious, and earnest in his desire to impart not only the mechanical manipulations of the art, but as well a knowledge of the principles involved. His knowledge of the subjects included in the chair of Mechanical Dentistry and Dental Metallurgy was probably not exceeded, if indeed equaled, by any other in the profession. His lectures, if written out, would form a more thorough treatise on these topics than is elsewhere to be found.

As a man, he was honorable, generous, genial, sympathetic; always ready to impart knowledge to those who asked, but remarkably modest and unassuming. He did not appear anxious to make friends or to secure confidence, but those who once learned to trust him never had cause to regret it. The confidence he won he maintained to the end. He occupied and deserved a high position in his profession, and will be long and kindly remembered."

